# The Alveolar Variant of Lobular Carcinoma and Its Mimickers: A Case Series

**DOI:** 10.7759/cureus.63909

**Published:** 2024-07-05

**Authors:** Habibat F Kolawole, Harpreet Rai, Peter Lovrics, Pooja Vasudev

**Affiliations:** 1 Department of Pathology and Molecular Medicine, McMaster University, Hamilton, CAN; 2 Department of Pathology, University of Toronto, Toronto, CAN; 3 Department of Surgery, St. Joseph's Healthcare, Hamilton, CAN

**Keywords:** beta-catenin, lobular breast cancer, alveolar variant, myoepithelial markers, lobular breast carcinoma, immunohistochemistry (ihc), breast cancer, diagnostic pitfalls, invasive lobular carcinoma of the breast

## Abstract

Invasive lobular carcinoma (ILC) is the most common special type of invasive breast cancer (IBC), accounting for 5-15% of IBCs. The distinct histomorphology of ILC reflects a special tumor biology, the hallmark of which is the lack of E-cadherin expression. However, the occasional presence of E-cadherin expression and the presence of IBC of no special type (IBC, NST)-like morphologies in ILC and vice versa make the diagnosis challenging.

We present two cases of the alveolar variant of ILC, a diagnostically challenging entity.

The first case is an 81-year-old female with two discrete right breast masses at 1 o'clock and 9 o’clock positions.

The second case is a 61-year-old female with two discrete left breast masses located at 11 o’clock and 12 o’clock positions. Core needle biopsies and subsequent mastectomy were performed in both cases.

On histology, three tumor foci were identified in the first case. The 1 o'clock focus showed IBC, NST, grade 3/3, ductal carcinoma in situ (DCIS) and lobular carcinoma in situ (LCIS). The 9 o’clock focus revealed ILC, classic and alveolar variants, grade 2/3, while a nearby third incidental focus was ILC, alveolar variant, both supported by lack of E-cadherin and β-catenin immunostaining.

The second case showed ILC, alveolar variant, grade 1 with LCIS component in the 11 o’clock lesion on both biopsy and mastectomy specimens. The lesion at the 12 o'clock position was diagnosed as IBC, NST, grade 2 with high-grade DCIS and LCIS components.

It is challenging to distinguish the alveolar variant of ILC from IBC, NST, and in situ lesions because of the overlapping morphology and occasional E-cadherin expression. Altered adherence of lobular cells may also be due to loss of α-, β-, and γ-catenins, and cytoplasmic re-localization of p120-catenin. Therefore, in ILC, the lack of β-catenin can be used as an adjunct along with E-cadherin. Myoepithelial markers such as p63 and smooth muscle myosin heavy chain (SMMHC) can be used to distinguish the alveolar variant of ILC from LCIS.

## Introduction

Breast cancer (BC) is recognized as a complex multifaceted disease with different biologic types and diverse natural history with varying spectrum of clinical, pathologic, molecular, prognostic, and therapeutic features [1]. Invasive lobular carcinoma (ILC) refers to a special type of BC with distinct histological and molecular features related to impaired cell adhesion [2]. It is the second most common type of BC after invasive ductal carcinoma (IDC) of no special type (NST) [3]. ILC is an invasive BC composed of dyscohesive cells that are often individually dispersed or arranged in a single-file linear pattern [4]. It is recognized as the most common special type of BC based on the World Health Organization (WHO) classification of tumors of the breast (5th edition) [4]. It accounts for 5-15% of all BC cases [5]. ILC is associated with bilaterality, multiple metastasis, older age at presentation (usually >50 years), lower histologic grade, higher pT stage, higher nodal stage, primary metastasis, and use of hormone replacement therapies, particularly those containing progesterone [2,6]. 

The WHO classification of tumors of the breast (5th edition) mentions four different ILC patterns (solid, alveolar, pleomorphic with apocrine or histiocytoid differentiation, and tubulo-lobular) [4,5]. The alveolar variant of ILC is a rare variant with only a few case reports describing the morphology in detail. Due to its unique morphology, this variant may be confused with IDC NST, atypical lobular hyperplasia (ALH), lobular carcinoma in situ (LCIS), or ductal carcinoma in situ (DCIS). Therefore, it is important to recognize this entity and distinguish it from its mimics due to clinical and therapeutic implications. 

We present two cases of the alveolar variant of lobular carcinoma with a detailed overview of its histological features and emphasize the importance of distinguishing it from close mimics. 

## Case presentation

Case 1 

An 81-year-old female with a history of diabetes mellitus, and renal and cardiac comorbidities presented with right breast discomfort of one-month duration. Breast ultrasound showed two distinct lesions, located in the retro-areolar position (1 o’clock) and mid-breast position (9 o’clock), respectively. The lesions were designated as BIRADS 5 (Breast Imaging-Reporting and Data System). These lesions were biopsied and a diagnosis of IDC NST, histologic grade 2 was made on histopathological assessment. 

She subsequently had a right simple mastectomy with sentinel and non-sentinel lymph node biopsies. Grossly, two distinct nodular lesions corresponding to the lesions identified on ultrasound were identified. They were 3.0 cm apart and separated by fibroglandular tissue. An additional third lesion located 1.1 cm posterior to the mid-breast (9 o'clock) lesion was identified. The retro-areolar (1 o’clock) lesion measured 2.6 cm in the greatest dimension with a solid gray-tan fibrotic appearance and ill-defined borders. It was 0.2 cm from the closest margin (posterior). 

The mid-breast (9 o’clock) lesion, located midway between the skin and the posterior margin of the right breast, measured 1.5 cm in greatest dimension with a stellate gray-tan fibrous cut surface. It was 1.5 cm from the closest resection margin (posterior). The additional third lesion located 1.1 cm posterior to the mid-breast lesion abuts the deep margin. It measured 1.1 cm in the greatest dimension with a firm, fibrotic nodular appearance.

Histologic evaluation revealed the presence of three distinct invasive breast carcinoma lesions. The retro-areolar (1 o’clock) lesion was diagnosed as a 2.6 cm IDC, NST pT2N0Mx, Nottingham histologic grade 3 with grade 3 DCIS and LCIS components. Lympho-vascular invasion and microcalcifications within both DCIS and invasive carcinoma components were identified. Immunohistochemistry showed positivity for estrogen receptor (ER) and progesterone receptor (PR) and equivocal human epidermal growth factor receptor 2 (HER-2) staining within the invasive carcinoma. Fluorescent in situ hybridization confirmed HER-2 negativity. 

**Figure 1:**
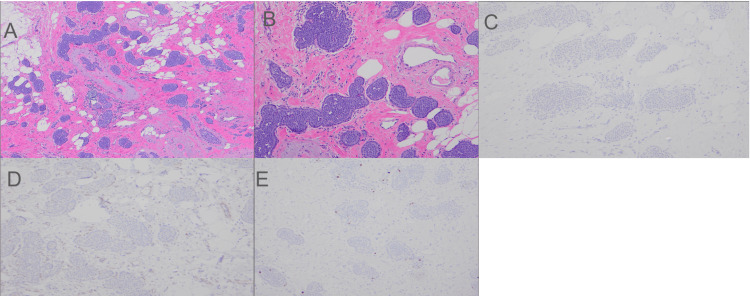
Photomicrograph showing histologic findings in Case 1. (A) ILC, alveolar variant from the mid-breast (9 o'clock) lesion showing infiltrative nests of low-grade invasive carcinoma cells (H&E stain ×40). (B) Higher power reveals round clusters of monomorphic rounded tumor cells with peripheral palisading (H&E ×100). (C) ILC, alveolar variant with negative E-cadherin stain (E-cadherin immunostaining ×100). (D) ILC, alveolar variant with negative beta-catenin (beta-catenin immunostaining ×100). (D) ILC, alveolar pattern with Ki-67 proliferation index  <1 % (Ki-67 immunostaining ×100). ILC, invasive lobular carcinoma; H&E, hematoxylin and eosin.

The mid-breast (9 o’clock) lesion was diagnosed as a 1.5 cm ILC (classic and alveolar variants) confirmed by a complete lack of E-cadherin and beta-catenin immunostaining (Figure 1), Nottingham histologic grade 2. Ki-67 proliferation index was less than 1% (Figure 1). Biomarkers showed positive hormone receptors (ER and PR) while HER-2 was negative (score 1+) by immunohistochemistry.

The additional third lesion was diagnosed as a 1.1 cm ILC, alveolar variant, Nottingham histologic grade 2. This was confirmed by a complete lack of E-cadherin immunostaining. The biomarker profile was similar to the second focus described above. Sentinel and non-sentinel lymph node biopsies showed benign lymph nodes with reactive changes and no evidence of metastatic carcinoma.

She subsequently received hormonal therapy with letrozole and declined radiotherapy due to multiple co-morbidities and is doing well with no evidence of recurrent or metastatic disease from an oncologic standpoint 38 months from the date of surgery.

Case 2****

A 61-year-old female presented with two discrete masses in the left breast. She has a past medical history of right breast IDC, NST managed with neoadjuvant chemotherapy, right mastectomy, post-surgical radiation, and tamoxifen therapy. Mammography of the left breast showed two discrete masses 0.5 cm apart, located at the 11 o’clock position, 3 cm from the nipple, and 12 o’clock position, 5 cm from the nipple, respectively. She had initial core needle biopsies of both lesions and subsequent simple left mastectomy with sentinel lymph node biopsy. 

Mastectomy revealed two grossly distinct lesions corresponding to the mammography findings. The lesion at the 11 o’clock position measured 1.0 cm in greatest dimension with a firm, tan, spiculated appearance and a central biopsy cavity. At the 12 o’clock position, the lesion was firm, yellow-tan, with ill-defined borders measuring 1.6 cm in the greatest dimension. 

**Figure 2:**
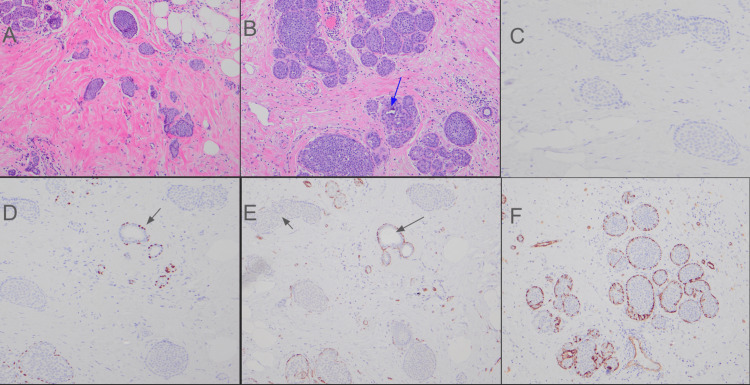
Photomicrograph showing histologic findings in Case 2 (A) ILC, alveolar variant, showing nests of cells with vague peripheral palisading (H&E ×100). (B) LCIS showing nests of cells lined by myoepithelial cells and occasional microcalcification (blue arrow) (H&E ×200). (C) ILC, alveolar variant, with loss of E-cadherin (E-cadherin ×100). (D) ILC, alveolar variant, with loss of myoepithelium around invasive component, while being maintained around the LCIS (black arrow) (p63 immunostaining ×100). (E) ILC, alveolar variant, with loss of myoepithelium around invasive component (short black arrow) while being maintained around the in situ LCIS component (long black arrow) (SMMHC immunostaining ×100). (F) LCIS with myoepithelium around a nest of cells (SMMHC immunostaining ×100). ILC, invasive lobular carcinoma; H&E, hematoxylin and eosin; LCIS, lobular carcinoma in situ; SMMHC, smooth muscle myosin heavy chain.

Histologic diagnosis of ILC, alveolar variant, preliminary grade 1 with LCIS component in the 11 o’clock lesion was made on both biopsy and mastectomy specimens. The invasive carcinoma cells of the alveolar variant of ILC form rounded clusters with vague palisading of tumor cells around the edges, but lack any true myoepithelial lining as highlighted by p63 immunostaining (Figure 2). LCIS, a close mimic of ILC, alveolar variant, on the other hand, shows a distinct flattened layer of myoepithelial cells with spindle-shaped nuclei (Figure 2). Breast biomarkers showed positivity for ER and PR and negative HER-2 (1+).

The lesion at the 12 o'clock position was diagnosed as IDC, NST, preliminary grade 2 with high-grade DCIS and LCIS components. There was no evidence of lympho-vascular invasion and lymph node or skin involvement and all margins were negative for tumor involvement. The findings were confirmed by immunohistochemistry showing loss of E-cadherin and beta-catenin. Loss of the myoepithelial layer was demonstrated by lack of p63 and smooth muscle myosin heavy chain (SMMHC) expression around the invasive carcinoma cells (Figure 2).

The four sentinel lymph nodes examined histologically showed benign reactive changes with no evidence of metastatic carcinoma.

She received treatment with adjuvant anastrozole postoperatively and is currently doing well 34 months post-surgery with no evidence of recurrence or metastatic disease. 

## Discussion

Alveolar ILC is a rare variant of ILC first described by Martinez and Azzopardi in 1979 when they described it as an “attempt to reproduce'' acinar structures or a “phase” before ILC cells finally lose cohesion [7]. A study by Dixon et al. described it to occur in women between 33 and 81 years (mean age of 59.9). Similar to both our cases, they described it as having a more favorable prognosis compared to the solid variant of ILC [8].

ILC is often diagnosed at an advanced stage and the patients tend to be three years older at diagnosis in comparison to IDC [6]. This finding correlated with our study as both patients are above the age of 60. ILC is difficult to detect on physical examination, and standard imaging techniques including mammography and magnetic resonance imaging have been reported to have better sensitivity in the detection and characterization of ILC [9]. 

There has been an increased incidence of ILC over the last two decades, particularly among post-menopausal women. This has been attributed to improved diagnostic techniques and the use of hormone replacement therapies, a relationship that has been stated to be a factor underlying its positivity for ER [2,10]. ILC poses unique challenges in terms of histologic diagnosis, management, and systemic treatment due to its distinct biologic profile, broad morphologic spectrum, and rarity of some of its variants including the alveolar variant which can be easily confused with mimics such as low-grade IDC, ALH, and LCIS [6,11]. 

The hallmark feature of ILC is the loss of E-cadherin, a tumor suppressor gene involved in cell-cell adhesion, resulting in the solid growth of small, dyscohesive cells, individually dispersed cells, or single-file strand architecture [2,8]. However, other cell adhesion molecules such as alpha, beta, and gamma catenins and p-120 catenin (proteins known to complex with E-cadherin) may be the altered molecules leading to loss of cell-cell adhesion [2,4]. Loss of E-cadherin is not diagnostic of ILC, and its loss can be seen in some cases of IDC and high-grade basal-like (non-lobular) BCs, presumably during the later stages of tumor progression [2]. In addition, approximately 15% of lobular lesions retain E-cadherin expression but with an aberrant (cytoplasmic) staining pattern, which should not be interpreted to exclude a lobular phenotype. Thus, it is important to interpret the immunohistochemistry in conjunction with detailed histologic analysis [2,12]. In addition, most cases of ILC with aberrant E-cadherin staining display evidence of impaired integrity of the cadherin-catenin complex with cytoplasmic rather than membranous staining for p-120 catenin [12]. The WHO classification of tumors of the breast (5th edition) mentions different ILC variants (solid, alveolar, pleomorphic, and tubulo-lobular) [4]. There exists great diversity in these variants, and they can be identified by either their cytologic features or architectural growth patterns. 

Histologically, the alveolar variant of ILC consists of small- to medium-sized, uniform, dyscohesive tumor cells with mild nuclear atypia and slight hyperchromasia typically growing in groups of at least 20 cells, forming globular aggregates separated by thin bands of collagenous fibrosis [2,8]. Sometimes, the outermost layer seems to have a peripheral palisading and slight dyscohesion from the central mass of cells, hence the term ‘alveolar.’ It can easily be mistaken for a focus of LCIS. However, a distinguishing feature is that ILC, alveolar variant, lacks surrounding myoepithelial cells. Thus, it is invasive and will be negative for myoepithelial markers including p63, calponin, and SMMHC, as demonstrated by our images for Case 2. The alveolar variant of ILC differs from IDC as there is a complete lack of gland formation in the former, and the diagnosis can be supported by immunohistochemistry demonstrating a lack of E-cadherin and beta-catenin staining. Polymorphous adenocarcinoma is another cause of diagnostic pitfall; however compared to the alveolar variant of ILC, it is triple-negative, BCL-2 positive, and E-cadherin-positive, and often has an aggressive course in young patients [2]. 

ALH and LCIS are both forms of non-invasive lobular neoplasia characterized by the proliferation of small, dyscohesive cells originating in the terminal duct lobular units (TDLUs), with or without pagetoid involvement of terminal ducts. However, ALH involves fewer than half of the acini in a TDLU, whereas in LCIS, greater than 50% of the acini in a TDLU are filled and expanded by the neoplastic cells. These lesions differ from ILC by the presence of myoepithelial cells [2,4]. 

The treatment of ILC is based on endocrine therapy following surgery to which great response has been reported [11]. This appears in line with the good outcomes of both our cases. Systemic chemotherapy is often associated with poor outcomes and low rates of complete pathologic response [6]. A better understanding of the unique molecular alterations and biologic profile of ILC and its subtypes is required for improvements in diagnosis, management, and overall survival. 

## Conclusions

The alveolar variant of ILC is a unique variant of ILC that has only been described in a few case reports. Morphologically, it is characterized by monotonous, alveolar-like globular clusters of low-grade carcinoma cells with peripheral palisading, rather than the usual single filing or isolated cells commonly associated with ILC, classic type. Due to this unique morphology, it can be misdiagnosed as LCIS, DCIS, or low-grade IDC, NST due to overlapping morphology and E-cadherin expression. These entities all have different treatment modalities and prognostic implications. Hence, it is important to correctly recognize and diagnose them accurately. The application of morphological criteria, myoepithelial markers, and beta-catenin immunostaining can help establish a diagnosis of the alveolar variant of ILC.
